# Effect of School-Based Educational Interventions on the Knowledge of Malaria and Dengue Among Higher Secondary School Children in Chennai, India: A Pre and Post-intervention Study

**DOI:** 10.7759/cureus.26536

**Published:** 2022-07-03

**Authors:** Chokkara Roja, Avudaiappan Seetha Lakshmi, M Anitha Rani, Alex Eapen

**Affiliations:** 1 Community Health Nursing, Sri Ramachandra Institute of Higher Education and Research, Chennai, IND; 2 Nursing Foundation, Sri Ramachandra Institute of Higher Education and Research, Chennai, IND; 3 Community Medicine, Sri Ramachandra Institute of Higher Education and Research, Chennai, IND; 4 Field Unit, Indian Council of Medical Research-National Institute of Malaria Research, Chennai, IND

**Keywords:** school-based interventions, school children, knowledge, control, prevention, dengue, malaria, health education

## Abstract

Introduction: School children are a means to reach and sensitize the community on the prevention of seasonal diseases such as malaria and dengue. The current study aims to determine the impact of school-based educational interventions on the knowledge of students toward the prevention and control of malaria and dengue in higher secondary schools.

Methods: This pre- and post-intervention study was conducted in three higher secondary schools in Zone IV, North Chennai, from September to December 2021. A total of 284 students in the age group of 13-17 years participated in the study. School-based educational interventions were delivered through PowerPoint-assisted lectures, participatory group activities, and demonstration of mosquito larvae and their control. The impact of the interventions as the change in knowledge level was analyzed using McNemar’s test, with a p-value of <0.05.

Results: Educational interventions led to the improvement in knowledge about malaria symptoms, such as fever (43.7% to 76.1%; p<0.001), chills (45.1% to 82.4%; p<0.001), and headache (46.1% to 86.6%; p<0.001), and the knowledge of *Aedes* mosquito bites as the cause of dengue transmission enhanced (41.9% to 92.2%; p<0.001). Similarly, there was an increase in knowledge on the identification of vector mosquito breeding sites inside the house (11.9% to 67.9%; p<0.001) and outside the house (10.9% to 69.7%; p<0.001) and mosquito net usage (21.5% to 76.1%; p<0.001) after the interventions.

Conclusion: School-based educational interventions had a significant impact on enhancing the knowledge on the prevention and control of malaria and dengue among school children. Involving school children can strengthen existing malaria and dengue prevention and control strategies in endemic areas.

## Introduction

Globally, 80% of the population is at risk of one or more vector-borne diseases [1]. Malaria and dengue are the major mosquito-borne diseases in the Southeast Asian region transmitted by the genus *Anopheles* and *Aedes*, respectively. These are co-endemic diseases with fever as a common symptom and have a significant influence on the community’s socioeconomic status [2]. Worldwide, in 2020, 241 million malaria cases were reported, which is higher compared to the 2019 statistics. This is due to disruption in malaria diagnosis, treatment, and preventive services during the COVID-19 pandemic [3]. Global climate changes and social inequalities within countries to access malaria treatment and prevention leave children and families in poor communities more vulnerable [4]. Dengue outbreaks have rapidly increased in several countries over the last two decades. The additional burden due to the COVID-19 pandemic on the healthcare system places the urban population at risk of dengue and other vector-borne diseases [5]. In the Southeast Asian region, dengue cases have increased by 46% from 2015 to 2019 [6]. In India, dengue cases increased from 101,192 in 2018 to 123,106 in 2021 [7,8]. Nevertheless, in Tamil Nadu, 615 malaria and 2,875 dengue cases were reported in 2021, out of which Chennai accounts for 75% of malaria cases [9].

In India, malaria and dengue are seasonal epidemic diseases that occur every year and are currently challenging to manage due to the COVID-19 pandemic restricting active surveillance and vector control activities. Community awareness and inter-sectoral coordination are needed to drive away these diseases [10]. In this context, India is driving its efforts toward malaria elimination after the launch of the National Framework for Malaria Elimination in 2016 and the National Strategic Plan for Malaria Elimination in 2017 with the vision of a malaria-free country by 2027 and elimination by 2030 [11].

Schools are one of the pivotal parts of the community that can serve as an important delivery mechanism in imparting malaria awareness for both children and their families [12]. According to the Ayushman Bharat school health program guidelines, trained students can act as health and wellness messengers in society to improve health practices [13]. Engaging school children as health messengers through a participatory approach is an important step as they bring drastic changes in the behavior of the community for the prevention and control of malaria and dengue [14,15].

The educational methods used to encourage participation by the students such as audiovisual aids, posters, and pamphlets are found to be appropriate and significant to empower the students to take care of their health and to make the right choices of healthy behaviors [13]. Evidence reported that school-based interventions facilitated through PowerPoint lectures, awareness programs, and demonstrations were effective channels to create awareness of malaria and dengue in the community through schools [16,17]. In India, very few studies have evaluated the effectiveness of school-based interventions by delivering lectures or participatory methods for the prevention of malaria and dengue [18,19]. So, the current study was undertaken to evaluate the impact of school-based educational interventions using both lectures and participatory methods on malaria and dengue prevention and control at selected higher secondary schools in Chennai, India.

## Materials and methods

**Figure 1:**
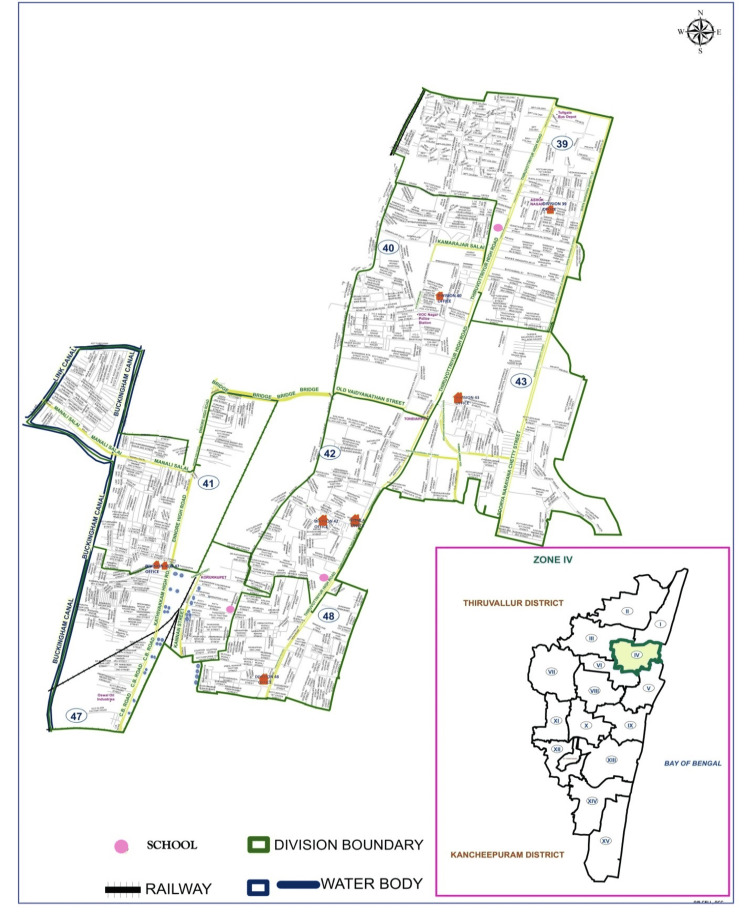
Map of Zone IV in Chennai city indicating the location of the schools

This intervention study was conducted in malaria- and dengue-endemic areas of Greater Chennai Corporation (Zone IV) in North Chennai, from September to December 2021. Zone IV covers a total population of 626,273 with 15 divisions. Figure 1 shows the study areas, namely, Old Washermenpet, Tondiarpet, and New Washermenpet. In total, 38 higher secondary schools were present in the study areas, out of which two government and one private school gave their consent to participate in the study. The students in the age group of 13-17 years and belonging to grades 9, 10, 11, and 12 were selected based on the inclusion criteria. In each grade, students from one section were selected based on willingness to participate in the study.

The sample size was computed using the nMaster software version 2.0 (Christian Medical College, Vellore, India). We assumed that the baseline knowledge of the study population on malaria and dengue was 50%, and the anticipated proportion of knowledge after the intervention was 60%. For precision 5%, power 90%, and 95% confidence interval, the minimum estimated sample size was 259. Assuming a 10% nonresponse rate, the sample size was increased to 284.

Heath education module was prepared in consultation with experts and science teachers. In each school, the following interventions were conducted to train the students. Initially, classroom teaching was done using PowerPoint lectures on malaria and dengue, including causes, modes of transmission, risk factors, mosquito breeding sites, symptoms, treatment, prevention, and control measures. Furthermore, in each class, students were divided into four groups with 8-10 members and motivated for group activities such as preparation of models, elocution, essay writing competition, posters, and preparation of charts. The one-day exhibition was conducted in each school, and the winners of the group activities were rewarded with prizes as a token of appreciation. All interventions were performed in the schools within three months (September to November 2021).

The self-administered questionnaire was prepared from malaria and dengue standard reference guides, information series, literature, and suggestions from professionals and was translated into the local language (Tamil) from the original version in English. The tools consisted of sociodemographic variables; 16 questions on malaria and 11 questions on dengue regarding mode of transmission, causes, risk factors, common symptoms, complications, and treatment; and eight questions on the identification of vector mosquito breeding sites and prevention of both diseases. The tools were validated among 20 students from a school that was not involved in the main study through concurrent validity. The researcher gave instructions about the tools to the students, and their responses were obtained before and after the intervention.

Data were cleaned, checked for completeness, and analyzed using SPSS version 20.0 (IBM Corp., Armonk, NY, USA). Descriptive statistics were used to calculate background variables, and McNemar’s test was performed to interpret the differences in proportion between pre- and post-intervention knowledge scores. A p-value of <0.05 was considered statistically significant.

The study received school permissions from the education department E.D.C.NO.A3/06977/2020 and Joint Commissioner (Health), Greater Chennai Corporation, Public Health Department H.D.C.NO.C6/3325/2021, Tamil Nadu. The study has obtained approval from the Institutional Ethics Committee (IEC) of Sri Ramachandra Institute of Higher Education and Research, Chennai (IEC-NI/20/OCT/76/106). Before data collection, written informed consent was obtained from the parents, and assent was obtained from each student. The study findings were disseminated to the research participants and school principals and officials.

## Results

The mean age of the study participants was found to be 15±1.6 years, and 170 (60%) were females. Class-wise distribution of students from grades 9, 10, 11 and 12 were 80 (28.2%), 60 (21.1%), 81 (28.5%), and 63 (22.2%), respectively. The respondents were predominantly from government schools (211 (74%)). The majority (147 (52%)) of the parents had <10,001 INR (modified Kuppuswamy socioeconomic scale) as a monthly family income; 110 (38.8%) were either illiterate or had a primary school education; 135 (47.5%) were skilled, shop, and market sales workers; and 123 (43.3%) had the source of information through television/mobile/Internet messages about malaria and dengue (Table 1).

**Table 1:** Sociodemographic characteristics of the school children (n=284)

Variable	Frequency (%)
Mean age (years)	15±1.6
Gender	
Male	170 (59.9)
Female	114 (40.1)
Grade	
Grade 9	80 (28.2)
Grade 10	60 (21.1)
Grade 11	81 (28.5)
Grade 12	63 (22.2)
Type of school	
Public	211 (74)
Private	73 (26)
Education of the family head	
Postgraduate/profession/honors	5 (1.8)
Graduate	11 (3.9)
Intermediate/diploma	7 (2.5)
High school (9-10)	83 (29.2)
Middle school (6-8)	68 (23.9)
Illiterate/primary school (1-5)	110 (38.8)
Income per month (modified Kuppuswamy socioeconomic scale)	
<10,001 INR	147 (51.8)
10,002-29,972 INR	109 (38.4)
29,973-49,961 INR	18 (6.3)
49,962-74,755 INR	10 (3.5)
Occupation of father	
Professional	2 (0.7)
Technicians and associate professionals	14 (4.9)
Clerks	2 (0.7)
Skilled, shop, and market sales workers	135 (47.5)
Skilled agricultural and fishery workers	25 (8.8)
Craft and related trade workers	26 (9.2)
Plant and machine operators, assemblers	13 (4.6)
Elementary occupation	67 (23.6)
Source of information	
Television/mobile/Internet messages	123 (43.3)
Posters/leaflets/newspapers	86 (30.3)
Friends/neighbors/school	21 (7.4)
Health personnel/hospital	54 (19)

The post-intervention malaria knowledge scores of the students about the mode of transmission were enhanced to 259 (91.2%). A significant increase in participant knowledge of malaria symptoms was observed for fever (216 (76.1%)), chills (234 (82.4%)), and headache (246 (86.6%)). We also noticed that 215 (75.7%) subjects have recognized that children below five years were at risk for malaria, and 192 (67.6%) subjects perceived cerebral malaria as one of the complications of severe malaria after the health education (Table 2).

**Table 2:** Knowledge of the students about malaria (n=284)

Variable	Pre-intervention	Post-intervention	p-value
	Frequency (%)	Frequency (%)	
Modes of transmission			
Mosquito bite	84 (29.6)	259 (91.2)	<0.001
Eating contaminated food	107 (37.7)	14 (4.9)	<0.001
Drinking contaminated water	93 (32.7)	11 (3.9)	<0.001
Symptoms of malaria			
Fever	124 (43.7)	216 (76.1)	<0.001
Chills	128 (45.1)	234 (82.4)	<0.001
Headache	131 (46.1)	246 (86.6)	<0.001
Vomiting	44 (15.5)	227 (79.9)	<0.001
Fatigue	9 (3.2)	125 (44)	<0.001
Risk of malaria			
Migrant population	49 (17.2)	201 (70.8)	<0.001
Children <5 years of age	112 (39.4)	215 (75.7)	<0.001
Old-age people	76 (26.8)	192 (67.6)	<0.001
Pregnant women	51 (17.9)	166 (58.4)	<0.001
Don’t know	126 (44.4)	4 (1.4)	<0.001
Complications of severe malaria			
Cerebral malaria	60 (21.1)	192 (67.6)	<0.001
Severe anemia	20 (7)	158 (55.6)	<0.001
Liver damage	13 (4.6)	162 (57)	<0.001
Renal failure	41 (14.4)	196 (69)	<0.001
Don’t know	150 (52.8)	12 (4.6)	<0.001

There was an improvement in the knowledge levels of children about dengue-transmitting mosquitoes (41.9% to 92.2%; p<0.001) and the biting time of dengue mosquitoes (34.1% to 93.3%; p<0.001). The post-intervention knowledge of students about the symptoms of dengue has improved for high fever (77.1%; p<0.001), severe headache (87.7%; p<0.001), pain behind the eyes (79.2%; p<0.001), and joint pains (69.4%; p<0.001) (Table 3).

**Table 3:** Knowledge of the students about dengue (n=284)

Variable	Pre-intervention	Post-intervention	p-value
	Frequency (%)	Frequency (%)	
Dengue-causing mosquitoes			
Anopheles	98 (34.5)	13 (4.6)	<0.001
Aedes	118 (41.9)	262 (92.2)	<0.001
Culex	67 (23.6)	9 (3.2)	<0.001
Biting time of dengue mosquito			
Day time	97 (34.1)	265 (93.3)	<0.001
Night time	125 (44)	12 (4.2)	<0.001
Afternoon time	62 (21.8)	7 (2.5)	<0.001
Symptoms of dengue			
High fever	110 (38.7)	219 (77.1)	<0.001
Severe headache	114 (40.1)	249 (87.7)	<0.001
Pain behind the eyes	45 (15.8)	225 (79.2)	<0.001
Joint pains	95 (33.4)	197 (69.4)	<0.001
Skin rashes	23 (8.1)	112 (39.4)	<0.001
Warning signs of severe dengue			
Severe abdominal pain	19 (6.7)	212 (74.6)	<0.001
Bleeding gums	42 (14.8)	173 (60.9)	<0.001
Liver enlargement	29 (10.2)	162 (57)	<0.001
Persistent vomiting	62 (21.8)	218 (76.8)	<0.001
Rapid breathing	42 (14.8)	121 (42.6)	<0.001
Restlessness	41 (14.4)	114 (40.1)	<0.001
Don’t know	105 (36.9)	3 (1)	<0.001
Home management for dengue			
Drink plenty of liquids	40 (14.1)	207 (72.9)	<0.001
Take medication as prescribed	80 (28.2)	160 (56.3)	<0.001

The awareness of the students on the identification of vector mosquito breeding sites in the house has improved for flower pot trays with water (61.3%; p<0.001) and trays with water under the fridge (67.9%; p<0.001). Recognition of breeding sites outside the house or in peri-domestic areas has enhanced for roof gutters (50.3%; p<0.001), abandoned tires (69.7%; p<0.001), and coconut shells (74.6%; p<0.001) after the interventions (Table 4). 

**Table 4:** Knowledge of the students about mosquito breeding sites (n=284)

Variable	Pre-intervention	Post-intervention	p-value
	Frequency (%)	Frequency (%)	
Mosquito breeding in the house			
Flower pot trays with water	25 (8.8)	174 (61.3)	<0.001
Trays under the fridge with water	34 (11.9)	193 (67.9)	<0.001
Waste/garbage bins with water	84 (29.6)	203 (71.5)	<0.001
Water cans/buckets	56 (19.7)	151 (53.2)	<0.001
Mosquito breeding outside the house			
Roof gutters	28 (9.8)	143 (50.3)	<0.001
Discarded tires	31 (10.9)	198 (69.7)	<0.001
Coconut shells	73 (25.7)	212 (74.6)	<0.001
Open storage water tanks	52 (18.3)	175 (61.6)	<0.001
Broken/discarded water bottles	40 (14.1)	182 (64.1)	<0.001

**Figure 2:**
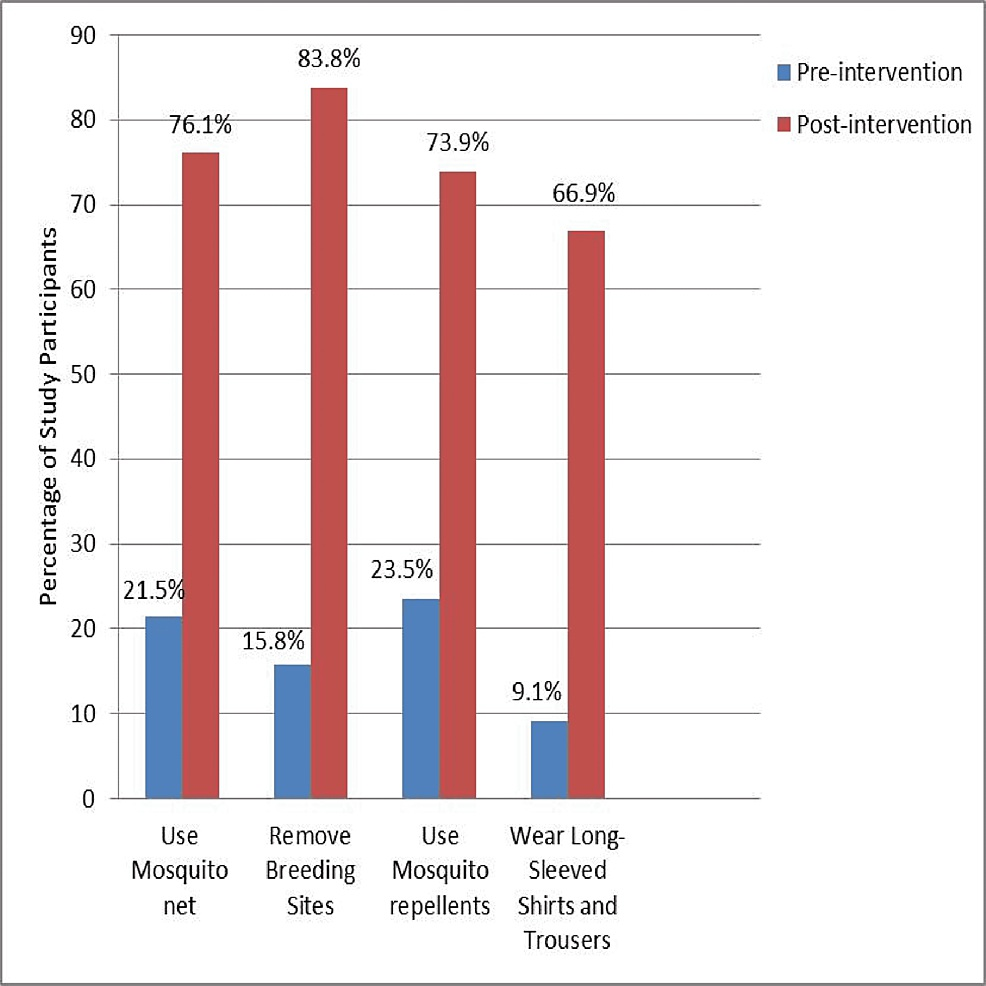
Knowledge of preventive measures for malaria and dengue

Children were able to recognize the preventive vector control measures for both malaria and dengue, such as using mosquito nets (21.5% to 76.1%; p<0.001), removing breeding sites (15.8% to 83.8%; p<0.001), using mosquito repellents (23.5% to 73.9%; p<0.001), and wearing full sleeve shirts and trousers (9.1% to 66.9%; p<0.001) (Figure 2).

## Discussion

This study highlights the significance of educational interventions adopted to sensitize the school children on malaria and dengue prevention. We observed a significant increase in the knowledge of school children on causes, modes of transmission, clinical symptoms, severe complications, and prevention of malaria and dengue.

Our study population reported television/Internet messages as the source of information on malaria and dengue. Earlier studies from Pondicherry, India, also showed that television was the major source of information for the students about mosquito-borne diseases [20]. Likewise, other countries such as Malaysia and Saudi Arabia also observed social media and mass media as important information sources for dengue prevention. This may be due to the fact that children are addicted to television and Internet messages [21,22].

Before the intervention, higher secondary school students believed that not only mosquito bites but also eating contaminated food and drinking polluted water were modes of transmission of malaria and dengue. After the health education, the majority of the students correctly identified the predominant cause of transmission, which is mosquito bites. Similarly, in Ghana, the cause of malaria was correctly identified through participatory health education programs [14]. Our study group reported that pregnant women and children below five years of age were at risk of malaria, whereas studies conducted in Ethiopia identified that pregnant women and children were vulnerable to malaria [15]. Further, in our study, school children’s knowledge of the symptoms of malaria has improved through classroom lectures, posters, and drawings. Similar findings were also reported from Nigeria, where classroom posters and drawings proved to be effective [23]. In the current study, students identified cerebral malaria as a complication of severe malaria. However, in Taiz, Yemen, students’ recognition of cerebral malaria was 51.1% after the health education [17].

Prior to the intervention, most of the inappropriate responses were observed on *Aedes* mosquitoes, their biting time, symptoms, and severe form of dengue, whereas after the delivery of classroom lectures and group activities, there was an increase in correct response rate with regard to dengue. Studies from Cambodia and Sri Lanka reported that the majority of school children were aware of the cause of dengue as “tiger mosquito,” and knowledge of its biting time, symptoms, and severe form of dengue improved with group discussions and health education [24,25].

In this study, the proportion of respondents’ awareness of mosquito breeding sites has improved through school health education. Previous studies from Karnataka, India, have observed that participatory health education enhanced the knowledge of school children on mosquito breeding sites [19]. Studies from Malaysia and Sri Lanka have been successful in raising school children’s knowledge of the identification of mosquito breeding sites through health education activities [21,25].

It is encouraging to observe that group activities enhanced the knowledge of high school children on the preventive measures of malaria and dengue, such as using mosquito nets, applying mosquito repellents, and wearing long-sleeved shirts and trousers. In South India, participatory health education enhanced the knowledge of school children on personal protective measures [19]. Evidence from Taiz, Yemen, revealed that educational interventions improved the knowledge of children on bed net usage and mosquito repellents as personal protective methods [17]. Classroom teaching activities with the demonstration of mosquito larvae and control methods, habitat identification, and prevention of vector-borne diseases have brought drastic changes in the knowledge of high school students [18]. Slideshows, wall slogans, and demonstrations sensitized school children toward malaria prevention [16,26]. Similarly, other studies from Zimbabwe and Thailand had used different health education methods to enhance the knowledge of school children about the prevention and control of malaria and dengue [27,28].

The strength of this study was the inclusion of 284 students from schools catering to a wide urban population in a malaria-endemic zone. Our study was designed with multiple health education approaches to motivate the school children in the prevention of seasonal diseases such as dengue and malaria. The current study is limited by the lack of a control group, and children below 12 years of age were not included due to the COVID-19 pandemic.

## Conclusions

School-based educational interventions had a significant impact on enhancing the knowledge of students on malaria and dengue prevention. The current study used multiple health education approaches such as classroom teachings with PowerPoint lectures and group activities on modes of transmission, symptoms, personal protective methods, identification of mosquito breeding sites, and preventive practices for malaria and dengue, which proved to be effective interventions. Health education interventions in schools have potential value to control seasonal diseases such as malaria and dengue as it improves communication among school teachers with the students and their family members.

## References

[1] (2022). World Health Organization: Global vector control response 2017-2030. https://apps.who.int/iris/bitstream/handle/10665/259205.

[2] (2022). World Health Organization: Dengue and malaria impacting socioeconomic growth. http://www.searo.who.int/mediacentre/releases/2014/pr1570/en.

[3] (2022). World Health Organization: World malaria report 2021: WHO global malaria programme. https://www.who.int/teams/global-malaria-programme/reports/world-malaria-report-2021.

[4] (2022). World Health Organization: Climate change and health. https://www.who.int/news-room/fact-sheets/detail/climate-change-and-health.

[5] (2022). World Health Organization: Dengue fact sheet. https://www.who.int/news-room/fact-sheets/detail/dengue-and-severe-dengue.

[6] (2022). World Health Organization: Regional office for South-East Asia: Dengue bulletin. https://apps.who.int/iris/handle/10665/340395.

[7] Bajwala VR, John D, Rajasekar D, Eapen A, Murhekar MV (2020). Burden of dengue with related entomological and climatic characteristics in Surat city, Gujarat, India, 2011-2016: an analysis of surveillance data. Am J Trop Med Hyg.

[8] (2022). European Centre for Disease Prevention and Control: Dengue cases reported worldwide. https://www.ecdc.europa.eu/en/dengue-monthly.

[9] (2022). National Health Mission: Health and family welfare department, Government of Tamil Nadu, India. https://nhm.tn.gov.in/en/nhm-programscommunicable-diseases/national-vector-borne-disease-control-programme-nvbdcp.

[10] (2022). COVID-19 Inter-Ministerial Notifications: Government of India: Guidelines for management of co-infection of COVID-19 with other seasonal epidemic prone diseases. https://covid19.india.gov.in/document/guidelines-for-management-of-co-infection-of-covid-19-with-other-seasonal-epidemic-prone-diseases.

[11] (2022). National framework for malaria elimination in India 2016-2030. https://nvbdcp.gov.in/WriteReadData/l892s/National-framework-for-malaria-elimination-in-India-2016%E2%80%932030.pdf.

[12] (2022). World Health Organization: Malaria prevention and control: An important responsibility of a health-promoting school. https://www.who.int/publications/i/item/malaria-prevention-and-control-an-important-responsibility-of-a-health-promoting-school.

[13] (2022). Operational guidelines on school health programme under Ayushman Bharat Health and Wellness Ambassadors partnering to build a stronger future. https://nhm.gov.in/New_Updates_2018/NHM_Components/RMNCHA/AH/guidelines/Operational_guidelines_on_School_Health_Programme_under_Ayushman_Bharat.pdf.

[14] Ayi I, Nonaka D, Adjovu JK (2010). School-based participatory health education for malaria control in Ghana: engaging children as health messengers. Malar J.

[15] Kebede Y, Abebe L, Alemayehu G, Sudhakar M, Birhanu Z (2020). School-based social and behavior change communication (SBCC) advances community exposure to malaria messages, acceptance, and preventive practices in Ethiopia: a pre-posttest study. PLoS One.

[16] Alok S, Nessa S, Ahil SB (2020). School training strategies for prevention and control of dengue. Indian J Community Med.

[17] Farea BA, Muharram AA, Baktayan NA, Assabri AM, Farea AA, Alsada MA (2020). Impact of health education on Kap towards malaria among basic schools pupils in Taiz Governorate. Republic of Yemen 2013: pre and post intervention study. Health.

[18] Swain S, Pati S, Pati S (2019). ‘Health promoting school’ model in prevention of vector-borne diseases in Odisha: a pilot intervention. J Trop Pediatr.

[19] Deepthi R, Naresh Kumar SJ, Prasanna Kamath BT, Rajeshwari H (2014). Participatory school health education on vector-borne diseases: engaging children as change agents. Int J Health Promot Educ.

[20] Jayanthi S, Vasudevan S, Raj P (2017). A study of the effectiveness of school health education programs on selected mosquito borne diseases: school based cross-sectional study. Int J Res Med Sci.

[21] AhbiRami R, Zuharah WF (2020). School-based health education for dengue control in Kelantan, Malaysia: impact on knowledge, attitude and practice. PLoS Negl Trop Dis.

[22] Usman HB, AlSahafi A, Abdulrashid O (2018). Effect of health education on dengue fever: a comparison of knowledge, attitude, and practices in public and private high school children of Jeddah. Cureus.

[23] Chukwuocha UM, Iwuoha GN, Ogara CM, Dozie INS (2020). Malaria classroom corner: a school-based intervention to promote basic malaria awareness and common control practices among school-age children. Health Educ.

[24] Khun S, Manderson L (2007). Community and school-based health education for dengue control in rural Cambodia: a process evaluation. PLoS Negl Trop Dis.

[25] Radhika NM, Gunathilaka N, Udayanga L, Kasturiratne A, Abeyewickreme W (2019). Level of awareness of dengue disease among school children in Gampaha District, Sri Lanka, and effect of school-based health education programmes on improving knowledge and practices. Biomed Res Int.

[26] Sharma VP (1987). Community-based malaria control in India. Parasitol Today.

[27] Midzi N, Zinyowera SM, Mutsaka MJ (2014). Impact of school based health education on knowledge, attitude and practice of grade three primary school children in Zimbabwe. J Community Med Health Educ.

[28] Okabayashi H, Thongthien P, Singhasvanon P (2006). Keys to success for a school-based malaria control program in primary schools in Thailand. Parasitol Int.

